# A Four Dimensional Spatio-Temporal Analysis of an Agricultural Dataset

**DOI:** 10.1371/journal.pone.0141120

**Published:** 2015-10-29

**Authors:** Margaret R. Donald, Kerrie L. Mengersen, Rick R. Young

**Affiliations:** 1 Mathematics and Statistics, University of New South Wales, Sydney, NSW, Australia; 2 Statistics Department, Queensland University of Technology, Brisbane, QLD, Australia; 3 Tamworth Agricultural Institute, NSW Department of Primary Industries, Calala, NSW, Australia; University of Vigo, SPAIN

## Abstract

While a variety of statistical models now exist for the spatio-temporal analysis of two-dimensional (surface) data collected over time, there are few published examples of analogous models for the spatial analysis of data taken over four dimensions: latitude, longitude, height or depth, and time. When taking account of the autocorrelation of data within and between dimensions, the notion of closeness often differs for each of the dimensions. Here, we consider a number of approaches to the analysis of such a dataset, which arises from an agricultural experiment exploring the impact of different cropping systems on soil moisture. The proposed models vary in their representation of the spatial correlation in the data, the assumed temporal pattern and choice of conditional autoregressive (CAR) and other priors. In terms of the substantive question, we find that response cropping is generally more effective than long fallow cropping in reducing soil moisture at the depths considered (100 cm to 220 cm). Thus, if we wish to reduce the possibility of deep drainage and increased groundwater salinity, the recommended cropping system is response cropping.

## Introduction

Where data are collected from a set of sites, at a series of time points, observations taken close to each other in either time or space may be autocorrelated. Highly autocorrelated observations reduce the number of effective observations, and statistical analyses and inferences which fail to take this autocorrelation into account are more prone to identification of erroneous significant relationships. In many applications, the spatial autocorrelations are the focus of interest, but in other applications, the aim is to account for them in order to obtain accurate and precise parameter estimates.

Spatio-temporal data are often analysed using models where spatial and temporal autocorrelation effects are separable, that is, with the assumption of no interaction between time and space. Cressie and Wikle [1] comment that separable covariance models “have very particular properties that are rarely seen in empirical studies of spatio-temporal dependence”.

A range of alternatives to address this drawback have been suggested in the more recent literature. Dynamical spatio-temporal models described in [1–3] utilise differential equations describing physical processes together with hierarchical models [4] involving a data model, a process model and a parameter model. The dynamical aspect of these models implies the possibility of a physical description. However, as Wikle and Hooten [5] note, substantial simplifications in the dynamics must often be made, with the redistribution kernels necessary for analytical solution not necessarily being representative of the data, and the assumption of homogeneity of the kernels over space and time being possibly unrealistic.

The aim of this paper is to evaluate a number of space-time modelling methods, which are described and compared in the context of a substantive case study of an agricultural field experiment designed to assess the impact of different cropping systems on soil moisture. The data arise from a balanced lattice design with different experimental treatments by plot. Earlier exploratory analyses of these data are given in [6, 7]. The dataset has a range of challenging features, including spatial autocorrelations that are not constant over time, nor over any of the three spatial dimensions. The model must therefore take account of both the spatial and temporal autocorrelations in the four dimensions of the data, the possible non-additive nature of the autocorrelation in time and space, and autocorrelations within the three-dimensional space ignoring time. The objectives of the data analysis were threefold: firstly, to estimate the contrast across time between the treatments of long fallow rotation and response cropping together with 95% credible intervals; secondly, to understand the time-varying nature of the contrasts; and thirdly, to find appropriate credible intervals for the contrasts when considered as time-series.

The primary spatial approach adopted in this paper is a conditional autoregressive (CAR) model [8, 9] with the proper priors of [10]. This model defines a local neighbourhood for the spatial domain, which improves computational efficiency since the precision matrix is sparse and repeated matrix inversions are not required. This is preferred to a kriging approach [11, 12] since kriging models are slow to converge in a Bayesian setting when datasets are large [13]. Moreover, [14] and [15] show an equivalence between the two types of model. This is supported by [16] and [17], who calibrate CAR models to kriging models. The primary temporal approaches include random walk and autoregressive models [9, 18–21] and penalised spline smoothers [22, 23].

Because of the complexity of the model developed to address the features of the data described above, the first modelling method fits a separate spatial model to each time period (date). The block updating Gibbs sampler of [7] is employed to provide estimates of the contrast of interest for each depth and day and their associated credible intervals. The second method takes these contrasts estimates and uses a time series model to describe their behaviour over time to gain insight into the time-varying process. In contrast to this two-stage approach, the third method fits a single structured additive model [8, 22] to the whole dataset. A feature of these approaches is that estimates can be smoothed across unequally spaced covariates and unequal time intervals.

## Data

The four dimensional data were soil water measurements from an agricultural field experiment. The field layout was a balanced lattice design comprising 4 replicate blocks of 9 treatments. Each plot was 15 m × 40 m, and measurements were taken from three neutron moisture meter access tubes (50 mm × 3 m deep installed 10 m apart along the centre of each plot. Thus, the four dimensional data consisted of soil water observations from 108 sites (defined across the surface), and taken at 15 soil depths from 20 cm to 300 cm, for 56 different dates spaced roughly monthly over a five-year period. The 108 measurement sites were arranged in 6 rows of 18 columns. Hence, data at each time point consisted of 1620 measurements at 108 sites (over 2 dimensions) at 15 depths, while the entire dataset consisted of 90,720 observations. The experiment was designed to determine a cropping system which would minimise water leakage below the crop root zone, and consisted of three cropping systems, running in different phases, and three perennial pastures, giving rise to 9 treatments. The first soil water measurements were made on June 26, 1995, and the last on April 27, 2000.

The treatments were

- Treatments 1–3: Phases of a long fallow wheat/sorghum rotation, where one wheat and one sorghum crop were grown in three years with an intervening 10–14 month fallow period. The 3 treatments were each of 3 phases of the long fallow 3 strip system.
- Treatment 4: Continuous cropping in winter with wheat and barley grown alternately.
- Treatments 5 and 6: Response cropping, where an appropriate crop (either a winter or a summer crop) was planted when the depth of moist soil exceeded a predetermined level.
- Treatments 7–9: Perennial pastures. The three treatments were lucerne (a deep rooted perennial forage legume with high water use potential), lucerne grown with a winter growing perennial grass, and a mixture of winter and summer growing perennial grasses.

The primary question for crop scientists was whether response cropping produced lower moisture values both at the intermediate and greater depths, in comparison with long fallow cropping, and whether this was sustained over different stages of the cropping cycle. This contrast was calculated as the average of treatments 1, 2 and 3 minus the average of treatments 5 and 6. The units of measurement for the contrast are log(neutron count ratio), a surrogate measure for moisture, with the higher neutron count ratios indicating wetter soils. Further details of the experimental design and aims are given in [24].

In modelling the key contrast evolving over time, the transformed variables log(rainfall+1), linear, quadratic and cubic time effects, together with interactions of year with sine and cosine terms with periods of a year and a half-year were considered as possible useful covariates.

### Ethics Statement

The cropping experiment was run on the private property ‘Hudson’, near Willow Tree, New South Wales, Australia, owned by Mr Robert and Mrs Edwina Duddy, with their full consent. No animals, endangered, protected or otherwise, were involved.

## Methods

### Subsection 1: Method 1

Let *y*_*tid*_ be the response variable measured on date *t*, at site *i* (of *S* horizontal plot sites), at depths from 20 cm–300 cm, indexed by *d* (*d* = 1,…,15). Let *j* be the treatment given at site *i*.

Method 1 fits a common spline model at each date, *t*, as follows:

$$\begin{array}{c} \begin{array}{c} y_{tid}=f_{tj}\left(d\right)+\psi_{tid}+\epsilon_{tid},\\ \epsilon_{tid}∼N\left(0,\sigma_{td}^{2}\right),\\ \psi_{tid}|\psi_{ti^{\prime }d},i\neq i^{\prime }∼N(\rho_{t}\sum_{i^{\prime }\in \partial i}\frac{\psi_{ti^{\prime }d}}{n_{i}},\frac{\tau_{td}^{2}}{n_{i}}),\\ f_{tj}\left(d\right)=X\beta_{tj}, \end{array} \end{array}$$ (1)

where *n*_*i*_ is the number of sites adjacent to site *i*, and *i*′ ∈ ∂*i* denotes that site *i*′ is a neighbour of site *i*. Neighbours are defined as the first order spatially closest observations at the same depth. This is the proper CAR model of [10], with *ρ*_*t*_ constrained to be common across all depths for a given date, *t*. **X** is the (common) design matrix for a set of spline basis functions across the 15 depths, while *β*_*tj*_ is the vector of coefficients for each day, *t*, and treatment, *j*. See [7] for details. The estimates from these fitted equations allowed the calculation of the contrasts of interest. From the treatment effects, the contrast of long fallowing versus response cropping was calculated at each depth as (*e*_1_ + *e*_2_ + *e*_3_)/3−(*e*_5_ + *e*_6_)/2, where *e*_*j*_ indicates the estimate for treatment *j* for a common depth, *d*. (Subscripts for depth have been omitted.) Thus, from Method 1, using [7], 56 treatment means at each depth (*d*) and date (*t*), i.e., 56 × 9 × 15 = 7560, and 56 × 15 (840) contrast estimates are estimated, together with their credible intervals.

Use of this method satisfies the first objective of the Introduction of estimating the key contrast together with credible intervals over time. However, it provides no insight into their time-varying nature.

### Subsection 2: Method 2

Here, time-series models were fitted to the contrast estimates of Method 1, This gives 15 time-series (one for each depth). We considered the time-series for the depths from 100 cm to 220 cm (focussing on the deeper depths, and 7 series in all). The set of 7 time-series can be modelled in two ways. The first alternative is as a multivariate time-series. However, in this case study the correlation structure of the contrasts was quite variable over the four dimensions. In particular, although there is some evidence of continuity in the contrasts across depths, it was unclear how to adequately model the depth-varying component of the corresponding multivariate random walk, or of a more general dynamic linear model. The second alternative is as a set of univariate time series, one for each depth. Since the depth is common for each time period, the subscript *d* is omitted from the equations below.

Three classes of time-series models were considered: regression models with time-varying covariates (Eq 2), random walk and autoregressive time-series models ignoring regression covariates (Eqs 3–5), and a combination of an autoregressive model with regression components using time-varying covariates and penalised spline models over time (Eq 6). The regression models accounted for covariates but assumed that the contrasts were independent over time. The autoregressive models ignored covariates but allowed for temporal dependence, although it was assumed that observations were equally spaced over time. The combined model provided a way of including covariates and accommodating unequal time periods. Furthermore, since these data may exhibit seasonality and perhaps trends, it was anticipated that the spline models might suggest further explanatory variables for the regression.

These classes of model are now described in more detail. Let *e*_*t*_ represent the contrast estimate at time, *t*.

The regression models are given by:

$$\begin{array}{c} \begin{array}{cc} e_{t}∼N\left(X\beta ,V\right), & 1/V∼Gamma(10^{-6},10^{-6}), \end{array} \end{array}$$ (2)

where **X** is a design matrix of time-varying covariates, namely log(rainfall+1) and interactions of year (as a factor) by sine and cosine terms with periods of a year and a half year.

The local level state-space model (random walk) of e.g., [18, 20, 21] was fitted.

$$\begin{array}{c} \begin{array}{cc} e_{t}=\mu_{t}+\nu_{t}, & \nu_{t}∼N\left(0,V\right),\\ \mu_{t}=\mu_{t-1}+\omega_{t}, & \omega_{t}∼N\left(0,W\right),\\ 1/V∼Gamma(10^{-6},10^{-6}), & 1/W∼Gamma(10^{-6},10^{-6}). \end{array} \end{array}$$ (3)

In a further version of this model, t-distributions with 10 and 4 degrees of freedom were substituted for the normal distributions for the observation and state errors, to allow for fatter tailed errors.

An alternative formulation of the random walk model which uses CAR neighbourhood models was also used. This formulation permits the neighbours to be weighted, and thus allows a correction for the unequal time intervals. Hence, weighted random walk models of order 1 (RW1) and order 2 (RW2) were fitted using [25].

$$\begin{array}{c} \begin{array}{c} e_{t}=\mu_{t}+\omega_{t}+\psi_{t},\omega_{t}∼N\left(0,V\right),\\ \psi_{t}|\psi_{t^{\prime }},t\neq t^{\prime }∼N(\sum_{t^{\prime }\in \partial t}\frac{w_{t^{\prime }}\psi_{t^{\prime }}}{w+},\frac{W}{w+}),\text{where}\\ w_{t^{\prime }}=1/\left|t-t^{\prime }\right|,\text{and}w+=\sum_{t^{\prime }\in \partial t}w_{t^{\prime }}, \end{array} \end{array}$$ (4)

with *V*, *W* defined as in Eq 3. The weight used is the reciprocal of the time difference between neighbours on the time scale (that is, the date difference).

The autoregressive models were fitted as follows [26, 27]:

$$\begin{array}{c} \begin{array}{cc} e_{t}∼N\left(\mu_{t},V\right), & 1/V∼Gamma(10^{-6},10^{-6}),\\ \mu_{t}=\alpha_{0}+\alpha_{1}Y_{t-1}, & \text{or},\\ \mu_{t}=\alpha_{0}+\alpha_{1}Y_{t-1}+X\beta , & \text{or},\\ \mu_{t}=\alpha_{0}+\alpha_{1}Y_{t-1}+\alpha_{2}Y_{t-2}, & \text{or},\\ \mu_{t}=\alpha_{0}+\alpha_{1}Y_{t-1}+\alpha_{12}Y_{t-12}. & \end{array} \end{array}$$ (5)

Note that the simple regression models using time-varying covariates (Eq 2), and the autoregressive models with time-varying covariates (Eq 5) were fitted to deal with the issue of a non-equally spaced time-series. The assumption of equally spaced contrasts across time in the models of Eqs 5 and 3 motivated the weighted random walk models of Eq 4. However, an alternative was to fit missing data models. This was done for the random walk of order 1 model (Eq 3) only. For these data, the highest common factor of the time intervals was 1, which gives a time-series of largely missing data (56 observed of 1768 observations or 3.2% non-missing observations). The missing data model was fitted for one depth only (140 cm).

The combined method involved fitting generalised additive models using penalised spline smooths over time [22, 28] using the software, BayesX (http://www.stat.uni-muenchen.de/ bayesx/bayesx.html). Let the contrast, *e*_*t*_, at date, *t*, and depth, *d*, be defined as

$$\begin{array}{c} \begin{array}{cc} e_{t}=f\left(t\right)+\epsilon_{t}, & \epsilon_{t}∼N\left(0,\sigma^{2}\right), \end{array} \end{array}$$ (6)

for each depth, *d*, with *f*(*t*) being fitted as a penalised spline over time with a random walk penalty of order 2. These models, like the regression model of Eq 2, do not account for autocorrelation over the time dimension, but they use the unequally spaced dates of the contrasts.

The time-series models of Method 2 capture the time-varying variance of the contrast, and while the contrast estimates themselves result from a model which accounts for spatial autocorrelation, the final models fail to reflect the spatial error. Hence, we experimented with precisions for the random walk model, which might reflect the full error which includes the within date variability of the contrast, in an attempt to satisfy objective 3 of the Introduction.

### Subsection 3: Method 3

Finally, using the full dataset, we fitted two additive structured models using the full 90,720 observations. The first model was defined as

$$\begin{array}{c} \begin{array}{cc} y_{tid}=\sum_{j}f_{j}\left(d\right)+\sum_{j}f_{j}\left(t\right)+\psi_{id}+\epsilon_{tid}, & \epsilon_{tid}∼N\left(0,\sigma^{2}\right),\\ \psi_{id}|\psi_{i^{\prime }d},i\neq i^{\prime }, & \psi_{id}∼N(\sum_{i^{\prime }\in \partial i}\frac{\psi_{i^{\prime }d}}{n_{i}},\frac{\tau_{d}^{2}}{n_{i}}), \end{array} \end{array}$$ (7)

The second model is analogous to that of Method 1 and was defined by

$$\begin{array}{c} \begin{array}{cc} y_{tid}=\sum_{t}\sum_{j}f_{j(t)}\left(d\right)+\psi_{tid}+\epsilon_{tid}, & \epsilon_{tid}∼N\left(0,\sigma_{t}^{2}\right),\\ \psi_{tid}|\psi_{ti^{\prime }d},i\neq i^{\prime }, & \psi_{tid}∼N(\sum_{i^{\prime }\in \partial i}\frac{\psi_{ti^{\prime }d}}{n_{i}},\frac{\tau_{td}^{2}}{n_{i}}), \end{array} \end{array}$$ (8)

Thus, for each timepoint, this model fitted a penalised curve for each treatment at each depth. It was, again, a layered model, which modeled site correlations using CAR models within each layer, and hence, allowed different variances at each depth and day, together with a final unstructured residual whose variance differed by day. Thus, the model from Eq 8 was again a considerable simplification of the model of Method 1. The CAR residual structure was the same, but was coupled with an unstructured variance common to each day, whereas the unstructured variance components of Method 1 (Eq 1) differed by date and by depth. As in Model 1, it fitted a series of penalised smooths across the depth dimension (by date and by treatment).

### Subsection 4: Priors

Priors for the Method 1 precisions for both structured and unstructured residual component precisions were Gamma(5, .005), with priors for the fixed coefficients being normal with mean zero and variance 10.

For the Method 2 models, which fitted the contrast across the time dimension, the priors for the coefficients were generally specified as a diffuse normal prior *N*(0, 10^6^). Priors for the precision terms of these models were initially set as Gamma(10^−6^, 10^−6^). However, almost all the models of Method 2 were rerun with priors for the precisions of Gamma(10^−4^, 10^−4^), and final model choice was made using models with this prior.

However, given that we wanted a meaningful temporal description of the contrast together with appropriate credible intervals, we experimented with various ways of apportioning estimates of the total error observed in the model from Method 1. Tables 1 and 2 give the settings for the 5 different priors used for the various models of Method 2, and Priors 3–5 show three schemes for apportioning the error.

**Table 1:** Various priors used for the precisions of the timeseries models of Method 2.

	*Precision for observational error*	Precision for random walk error*
Prior 1	∼ Gamma(.000001, .000001)	∼ Gamma(.000001, .000001)
Prior 2	∼ Gamma(.0001, .0001)	∼ Gamma(.0001, .0001)
Prior 3	mean *τ*	∼ Gamma(.000001, .000001)
Prior 4	*total* * *r*	*total* * (1−*r*)
Prior 5	∼ Gamma(.000001, .000001)	mean *τ*

*total* ∼ *Gamma*(*a*, *b*), *r* ∼ *Beta*(1, 1)

*Priors 1 & 2 were also used for other timeseries models. See the Priors Section.

**Table 2:** Various priors used for the precisions of the timeseries models of Method 2 (cont).

*Depth (cm)*	*Mean *τ**	*Gamma(a,b)*	
100	1395	6.934	.004971
120	1759	6.024	.003425
140	2241	12.413	.005538
160	3019	52.316	.017327
180	3226	87.249	.027045
200	3201	180.410	.056354
220	2175	82.412	.037894

*a,b* calculated via method of moments from mean *τ* & 95%CI for posterior in Method 1

Priors for the Method 3 models were set as the default BayesX software priors, with all precisions having a Gamma(.001, .001) prior.

### Subsection 5: Model Comparisons

We adopted the Deviance Information Criterion (DIC) of [29] as the method for model comparison, despite the deficiencies outlined in [30]. Thus, we planned to compare the the full models of Method 1 and Method 3 via the DIC, in addition to choosing a model from the many models of Method 2. Within the Method 2 models, only the models fitted using WinBUGS were compared.

When comparing random walk models of order one (See Results), we considered the root mean square of predictive error as a basis for model choice. This is defined as

$$\begin{array}{c} \sqrt{\sum {\left(y_{t+1}-E\left(y_{t+1}|y_{1},y_{2},...y_{t},\theta \right)\right)}^{2}}. \end{array}$$

### Subsection 6: Computational details

The model of Method 1 which produced the contrast estimates (also used by Method 2) was fitted using custom built software, pyMCMC [31], which used the Gibbs sampler of [7] with its block updating and linear splines. Its daily models had a 6,000 iterate burnin and 16,000 iterates in all. Fewer burnin iterates were needed because of the block updating.

The models of Method 2 were fitted using BayesX [32, 33] or WinBUGS [25]. The BayesX software was used because it offered penalised smooths over time, and because actual dates could be used in the fit. It was thought that such models would offer insight into the seasonality and/or trends in the data. WinBUGS was used because of its transparency and its robustness as a well established software.

The univariate time-series models of Method 2 were run with 2 chains, 120,000 iterates with a 100,000 burnin when using WinBUGS and Gelman-Rubin statistics were checked. This burnin was unnecessarily large. Models fitted using BayesX, Eq 6, were run with a 10,000 iterate burnin and 60,000 iterates in all (again probably unnecessarily large, given that this software uses block updating [28]).

Geweke diagnostics [34] for convergence and Raftery-Lewis estimates for accuracy [35] were checked and found to be satisfactory for all models, except where otherwise noted.

## Results

### Method 1

**Fig 1:**
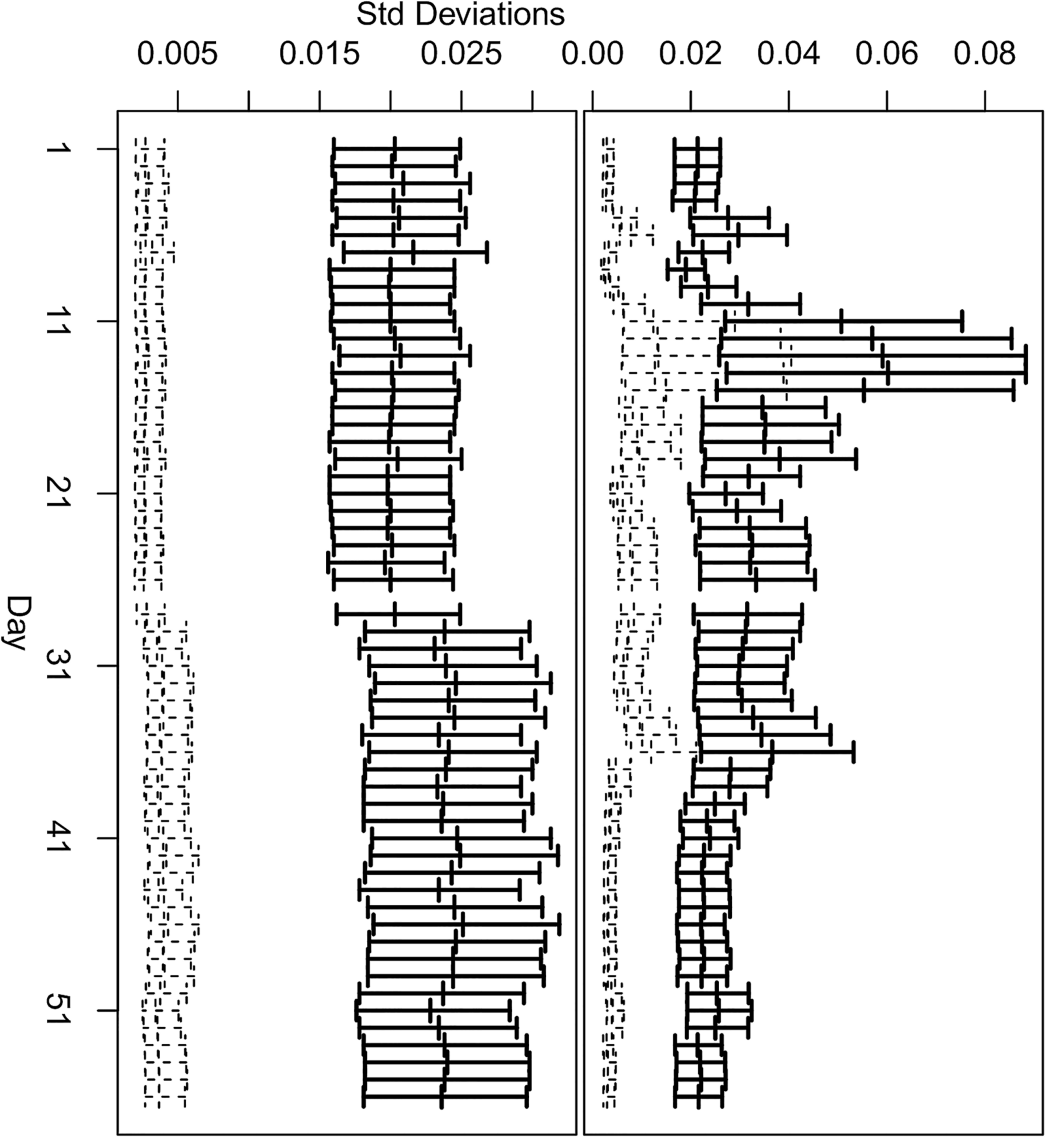
Method 1: Spatially structured and unstructured standard deviations. Top panel: and 95% credible intervals at depths 100 cm. Bottom panel: 95% credible intervals at depths 220 cm. The spatial standard deviations are shown using heavy black lines and bars, the unstructured standard deviations using dotted lines and bars.

**Fig 2:**
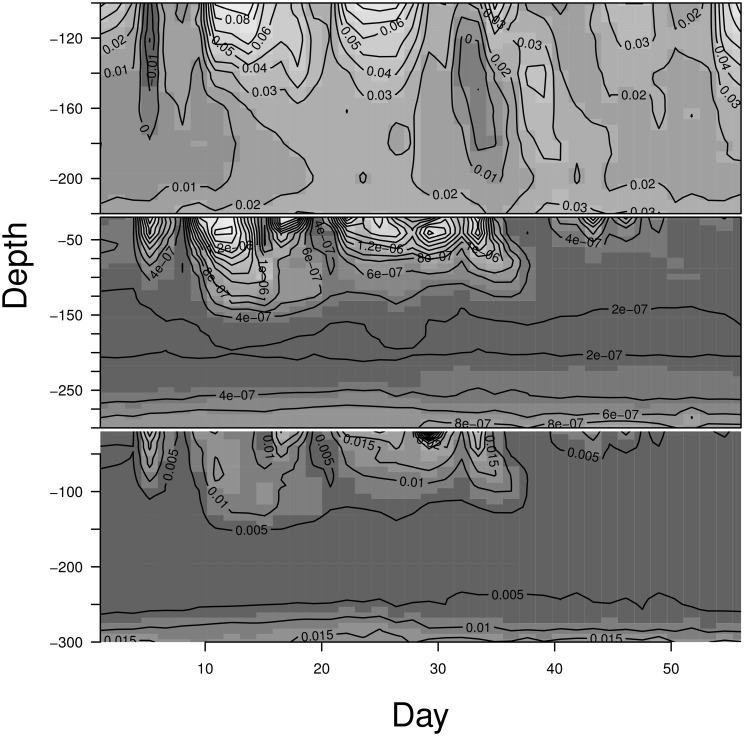
Method 1: Top panel—Long fallowing vs Response cropping. Contour graphs from the point estimates from the MCMC iterates. Middle panel—the square root of the unstructured variance components. Bottom panel—the square root of the spatial variance components. Method 1 model.

Not unexpectedly, the variances at the shallower depths show greater variability across the sampling dates, than those at greater depths. Fig 1 illustrates this, showing the square roots of the spatial and the unstructured variances for each date at depths 100 cm and 220 cm based on the model of Method 1. The comparable graphs across all depths show decreasing variability with increasing depth of these parameters across the sampling dates (Figs Y-AE in S1 File). This decreasing variability with depth is also seen in the contour smooth for the variance components across days and depth shown in the two lower panels of Fig 2. The variability in these parameters justifies the choice to fit the same model across all sampling dates thereby allowing the parameters of the original model to vary by date, since a description of their evolution across time was not obvious a priori.

The estimates for the contrasts at all depths and their 95% credible intervals from Method 1 satisfy the first objective of the analysis, and are given in the online supplement, as are graphs of the fits for the time-series from all seven depths (Figs A-X in S1 File).

**Fig 3:**
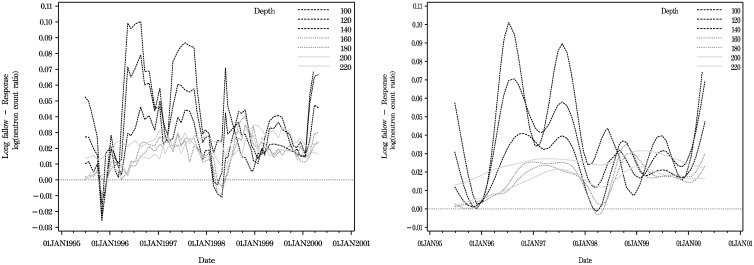
Long fallowing vs Response cropping at all depths. Left panel: Point estimates from the MCMC iterates of Method 1. Right panel: Spline curves from BayesX pspline estimation (Method 2, Eq 6).

The left panel of Fig 3 shows the point estimates from Method 1 for the contrasts at depths 100 cm to 220 cm. This graph exhibits an apparent continuity of the contrast estimates across time and depth. The same estimates are graphed again as a contour graph of moisture across day and depth (top panel Fig 2) in order to show the continuity across time and depth.

**Fig 4:**
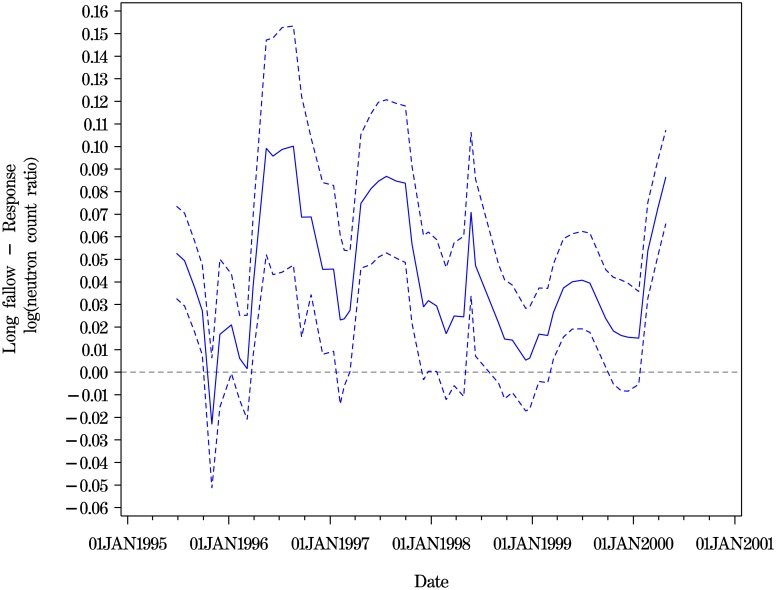
Long fallowing vs Response cropping at depth 100 for all trial dates. Point estimates and 95% CIs from MCMC iterates from Method 1.

**Fig 5:**
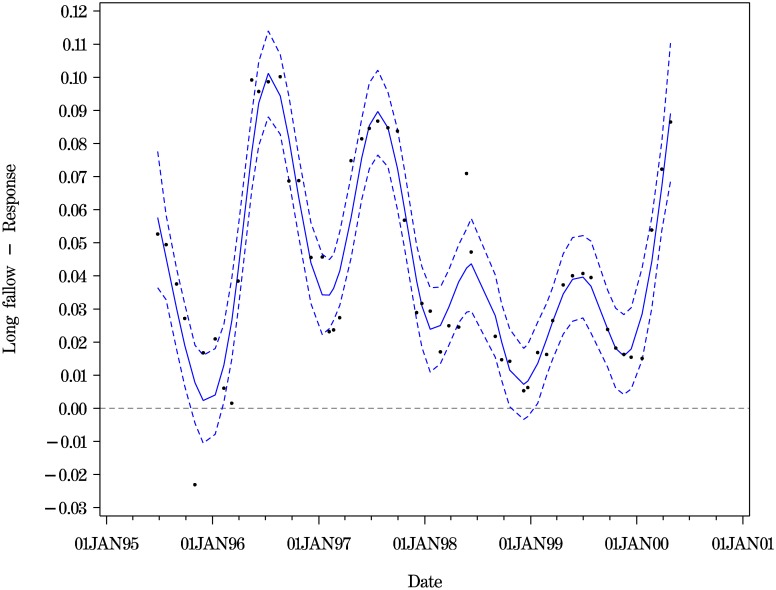
Long fallowing vs Response cropping at depth 100 for all trial dates. Penalised spline smooth across dates. Point estimates and 95% CIs (Method 2, Eq 6).

Two fits for the contrasts at depth 100 cm are shown (Figs 4 and 5). Fig 4 shows the fit for Method 1. The corresponding estimates can be seen to have much wider credible intervals compared to the time-series fits shown in S1 File and the penalised spline fit of Fig 5.

### Method 2

Fig 5 shows the penalised smooth from Method 3 Eq 8, and illustrates the seasonality observed in all models at the shallower depths. The 28 term regression fit from Eq 2 of Method 2 (shown in S1 File) echoes the penalised spline fit, but with the discontinuities expected in a model with interactions of year by periodics. The random walk of order two model of Method 2 (shown as Fig B in S1 File) is very similar. These three fits over time show comparable 95% credible intervals.

Comparisons of the time-series models of Method 2 are given in Table 3 for the contrast at depth 140 cm and were used to consider various models and ways of dealing with the unequal time spacing. Table 3 indicates that the AR(1) and AR(2) models are essentially equivalent, and that the AR (1)(12) model is a poor model (with its negative estimate for the number of parameters). Table 3 shows the DIC and pD varying for differing priors for the random walk models, but not varying for the AR models. This issue is discussed further in the Model Comparisons section.

**Table 3:** Summary of DICs for Contrast 1 (Long fallowing vs Response cropping) at Depth 140.

	*Prior 1*		*Prior 2*	
*Model*	*pD*	*DIC*	*pD*	*DIC*
Regression	30	−377		
AR(1)	4	−343	4	−343
AR(1)(12)	−2	−356	−2	−355
AR(2)	4	−343	5	−342
RW(1)	69	−435	36	−379
RW(1) (weighted)	73	−468	40	−392
RW(1) (t_10_ distribution)	73	−450	39	−378
RW(1) (t_4_ distribution)	74	−451	41	−375
RW(2)	20	−370	23	−373
RW(2) (weighted)	26	−390	43	−395
RW(1) (1768 time points)	49	−304	(Prior 5)	

Prior 1: both precision priors Gamma(0.000001,0.000001)

Prior 2: both precision priors Gamma(0.0001,0.0001)

Table 4 compares the models fitted using Eqs 2 and 5. This table shows that additional periodic covariates improve the fit of the AR1 model at depths of 100 and 120 cm, but for all other depths the simple AR1 model accounts adequately for the data without the need for rainfall or periodics. This is not surprising, since the time-series models take out the random shocks that might be explained by such terms. Note that the model ‘AR1+5’, a covariate model in combination with an AR1 model, posits (somewhat unrealistically) the same amplitudes across the years for the cyclical behaviour, but (realistically) posits a common time of year for the yearly maxima and minima. This table also gives the DICs for the regression model with interaction terms of year by periodics which permits differing amplitudes for different years, together with a cubic over time, giving 27 time covariates. Not surprisingly, with 28 parameters to fit 56 observations, these models from Table 4 show the regression model doing better than the AR1 models for all depths (except 200 and 220 cm, where they do equally well).

**Table 4:** DICs for Long fallowing vs Response cropping: 1st order autoregressive models vs simple regression model.

	AR1 With rainfall **		*AR1*		*AR1+5*		*Regression(28)*	
*Depth*	*pD*	*DIC*	*pD*	*DIC*	*pD*	*DIC*	*pD*	*DIC*
100	5	−279	4	−278	9	−289	30	−315*
120	5	−301	4	−303	9	−306	30	−344*
140	5	−341	4	−343	9	−342	30	−377*
160	5	−386	4	−386	9	−386	30	−425*
180	5	−414	4	−414*	9	−410	30	−433
200	5	−449	4	−450*	9	−444	30	−449
220	5	−457	4	−458*	9	−450	30	−455

* Better models

** Covariate: log(rainfall+1)

t *AR*1 + 5: Covariates: log(rainfall+1), sin(x), cos(x), sin(2x), cos(2x), x = date*2*π*/365

Regression(28): Covariates: x,x*x,x*x*x, year*(sin(x), cos(x), sin(2x), cos(2x)

Table 5 shows the DICs and the estimated number of parameters (pD) for the random walk models of order one and two, both weighted and unweighted. However, these models were not used in our final model comparisons, as we did not get stable solutions for the untransformed contrast variable, for reasons explained in the Model Comparisons section.

**Table 5:** DICs for Long fallowing vs Response cropping: random walk model comparisons, using Prior 2.

*RW1*			*RW1 (W)*		*RW2*		*RW2 (W)*	
*Depth*	*pD*	*DIC*	*pD*	*DIC*	*pD*	*DIC*	*pD*	*DIC*
100	47	−342	47	−346	21	−332	38	−340
120	39	−348	42	−360	26	−323	40	−360
140	36	−379	40	−392	23	−373	43	−395
160	34	−413	38	−424	25	−417	43	−419
180	32	−433	36	−434	25	−439	43	−419
220	28	−457	34	−448	24	−458	42	−424
220	28	−461	35	−452	24	−463	43	−427

(W): inverse time interval weights.

The random walk models of order two (RW(2)) are identifiable models, and when the contrast variable was standardised, gave estimates which were not dependent on the choice of priors. (However, the transformation of the variable meant that the DICs could not be compared with the models having the unstandardised response variable.) The RW(2) models allowed the calculation of the ratio (W/V) between the two types of variance in the model (Eqs 3 and 4 which is the signal to noise ratio [20]. The signal to noise ratio for the different depths is tabulated in Table 6 and shows that at the lower depths the observational error outweighs the signal.

**Table 6:** Square root of the Signal to Noise ratio for the RW models.

*Depth (cm)*	*SN ratio*	*95% CI*
100	1.06	(.58, 2.30)
120	.59	(.23, 1.09)
140	.63	(.19, 1.46)
160	.92	(.37, 2.10)
180	.71	(.29, 1.43)
200	.23	(.08, .48)
220	.13	(.05, 38)

The penalised spline fits from Eq 6 (right panel of Fig 3) show seasonal peaks and troughs which vary in amplitude across the years, thereby suggesting interactions of year by sine and cosine terms with periods of a year and half year. These curves also show the periodic behaviour dampening with increasing depth. We found significant terms in the rainfall and interactions of year by half-yearly periodic simple regression model, but such models showed the expected problems of sine curves being too smooth at their peaks and troughs with disjuncts at the year breaks, thus giving rise to serially correlated errors. We included these models because they gave a basis for comparison with the WinBUGS models where equality of time intervals was assumed, and allowed a possibility of a correction when used in combination with the AR models.

The missing data model, motivated by objective 3 in the Introduction, was based on Eq 3 since this was found to be close to the best model of those fitted. This was fitted because the approximation of equally spaced time intervals in the WinBUGS models seemed a considerable oversimplification. The graph for one missing data model, which attempted to construct credible intervals to reflect the spatial variability by adjusting the priors for the two variances of an RW1 model, is shown in Fig L in S1 File. This fit used prior 5 and the credible intervals for the missing values reflect the prior. Table 7 shows the apparently arbitrary nature of this undertaking, with the goodness of fits (as demonstrated by *R* ^2^) showing highly differing values depending on the prior. However, prior 3 allocates the total variance from Model 1 to the observational error (a very informative prior) and thus allows no variation to be absorbed by the random walk. This leads to the poor fit (*R* ^2^) and the low number of estimated parameters of Table 7 for this prior. Prior 4 partitions the total variance (estimated as a gamma prior) from Model 1 between the observational error and the random walk error. This leads to models with meaningful, but high, estimated numbers of parameters (pD). Based on the DIC, these models are poorer than the best models from Table 4. Prior 5 effectively allocates all of the error from Model 1 to the random walk component, forcing the observational error to be very small. This gives a an unsatisfactory ‘join the dots’ representation, signalled by the excessively large pD (greater than the number of degrees of freedom of the data). (The negative DICs also show that there is a problem with this model.) The issue of the error in RW(1) models is discussed further in the Model Comparisons section.

**Table 7:** *R* ^2^, *pD* and *DIC* for the RW(1) weighted models using priors 3–5.

	*Prior 3*			*Prior 4*			*Prior 5*		
*Depth (cm)*	*R^2^*	**pD**	**DIC**	*R^2^*	**pD**	**DIC**	*R^2^*	**pD**	**DIC**
100	33%	13	−255	80%	36	−258	99%	100	−411
120	23%	9	−277	79%	35	−279	99%	97	−421
140	12%	6	−299	80%	35	−271	99%	94	−433
160	16%	5	−323	83%	35	−251	100%	90	−446
180	19%	5	−332	85%	34	−246	99%	89	−448
200	27%	4	−337	86%	34	−239	99%	89	−448
220	18%	3	−318	82%	34	−225	99%	94	−434

As described in the Methods, we also compared models with respect to the root mean square predictive error. In particular, we compared the the RW(1) models under Prior 1 and Prior 2 (Table 8). This criterion shows no sensitivity to the choice of prior.

**Table 8:** Root mean square predicted error for RW1 models under Priors 1 & 2.

*Depth*	*Prior*	*Median*	*25%ile*	*75%ile*
100	Prior 1	0.020	0.019	0.020
100	Prior 2	0.019	0.019	0.020
120	Prior 1	0.016	0.015	0.016
120	Prior 2	0.015	0.014	0.016
140	Prior 1	0.011	0.011	0.011
140	Prior 2	0.010	0.010	0.011
160	Prior 1	0.0075	0.0073	0.0077
160	Prior 2	0.0073	0.0070	0.0077
180	Prior 1	0.0057	0.0054	0.0059
180	Prior 2	0.0056	0.0054	0.0059
200	Prior 1	0.0037	0.0035	0.0039
200	Prior 2	0.0041	0.0039	0.0043
220	Prior 1	0.0035	0.0033	0.0037
220	Prior 2	0.0039	0.0037	0.0041

Figs A–X in S1 File show the fits from Method 1, and for the time-series from Method 2, including the penalised smooths of Method 2, Eq 6. These show periodicity at the shallower depths decreasing with increasing depth. The RW(2) model fits (Method 2) are also shown.

*Convergence*

Tests for convergence (using Geweke’s test [34]) for the first model of Method 3 Eq (7) showed failure to converge. This is not surprising since this model is a major simplification of the model, Eq 1, from Method 1. See Methods. The more complex model from Method 3 Eq (8) also failed to converge. Hence, the modelling strategy of Method 3 was not pursued. However, the BayesX penalised spline models along the time dimension which modelled the 56 contrast estimates from Method 1 (Method 2, Eq 6) converged satisfactorily.

Models from Methods 1 and 2, with the exception of the random walk models, showed successful convergence using Geweke statistics [34] and Raftery-Lewis statistics [35] for the quantities of interest.

Table 4 indicates that the regression models with periodic terms are the better descriptions for the behaviour of moisture contrast over time at the shallower depths, while simple AR(1) models are sufficient to capture the damped periodicity at the deeper depths. Table 5 shows that the random walk of order 2 models give an almost equivalently good fit as the best models from Table 4. For all models and methods, it is the case that point estimates and 95% credible intervals for this contrast are generally positive, leading to the conclusion that long fallow cropping generally led to moister soils than response cropping. Thus, if we wish to reduce the possibility of deep drainage and increased groundwater salinity, the recommended cropping system is response cropping.

## Discussion

This paper has provided a careful evaluation of a range of spatio-temporal models for a real case study. Some remarks are now made about the choice of models in the context of the experimental design and about the inferences drawn about the problem itself.

The experimental design was a balanced lattice design. Two issues arise here. First, the models considered in this paper treated the data as observational since the observations reflect the randomisation inherent in the design. An alternative approach is to incorporate the design structure explicitly in the models, for example by fitting the replicates and obtaining an error term with respect to them. The two approaches have merit and are arguably equally appropriate. Second, even though the observations embody the design characteristics, the spatial character of the data is not necessarily adequately described by the randomisation and a subsequent design-based analysis. This provides further motivation for our modelling strategy, in which the replicates are dealt with as neighbours, so that in the CAR-layered model the replicate error forms part of the spatial error. The comparative characteristics and performance of the modelling methods considered in this paper are discussed in further detail below.

As a reviewer comments, an alternative modelling strategy would have been to fit the replicates and obtain an error term with respect to them. However, we feel that our modelling strategy, in which the replicates were treated as neighbours, is equally appropriate. Thus, in the CAR-layered model used, the replicate error formed part of the spatial error.

It should be noted that the comparisons are between systems and not crops: systems of long fallowing v. response cropping and so on, which have the potential to use soil water, or lose it as deep drainage depending on the crop planting and sequencing rules inherent in the design of each system. In this context, the crops are merely tools with which to suck soil water given the crop planting decision rules and rotational constraints (For example, disease carry over) which characterise each system. We take the crop rotations and differences in crops into account in that we have 9 treatments (although an opportunity crop in one year may differ from the opportunity crop on another year within a treatment, and this is what response cropping is all about—the farmer plants (or fails to plant) the most appropriate crop given the soil water status, time of year, and the potential for disease carry over from previous crops. The aim in managing the experiment was to grow a healthy crop appropriate for the system. A comparison between crops would be a separate exercise to this system comparison and would be fraught with confounding issues as the experiment was not designed for such a comparison.

The layered CAR analysis [6, 7] of these data showed that response cropping delivers lower moisture levels for most times of the year, in contrast to long fallow cropping. At the shallower depths, not surprisingly, this contrast exhibited considerable periodicity which attenuated with depth. It appears that the temporal component adds little additional uncertainty to the contrasts which has not been captured by model 1.

As discussed below, there are very few papers which deal with four-dimensional data, and frequently, the work is simply descriptive in one of the dimensions. The methods we used allowed us to fully explore the complexity of these four dimensional agricultural data.

### Modelling spatio-temporal data

Most spatio-temporal modelling deals with two spatial dimensions (surface) and time. Within the context of two spatial dimensions × time much work is largely descriptive. For example, Teschke et al [36] and Bell et al [37] use maps at several timepoints, with the maps essentially being descriptive devices. The more complex models are generally Bayesian and use either geostatistical methods or the convolution CAR prior of [8]. Models with CAR priors typically partition the error term in the model, *ϵ*_*it*_ as *e*_*i*_, *e*_*t*_ and *e*_*it*_, where the first two error terms capture the structured spatial random effects and the structured temporal random effects, and *e*_*it*_ is a simple unstructured random effect with *e*_*it*_ ∼ *N*(0, *σ* ^2^). See, e.g., [38–42], where the last three papers use BayesX software [32, 33] to conduct the analyses.

Assuncao et al [43] fit quadratics over time which differ for each spatial location, and for which the coefficients are smoothed using CAR priors, but both space and time are separable. This elegant solution to modelling very short time sequence data allows the possibility of seeing increasing and decreasing rates, while accounting for spatial closeness. Assuncao and others [44, 45] use space-varying regression coefficients on their quadratic models in time. Yan and Clayton [46] use the space-time interaction to define a set of space-time separable clusters carrying a specific risk, and fit a final unstructured random effect.

Abellan et al [47] decompose the error term into a structured temporal effect, a structured spatial random effect, and a time by space interaction random effect which is a mixture of two Gaussians and thus equivalent to an outlier or contaminant model. This allows the identification of sites (areas) and times which fail to fit the common temporal and common spatial patterns.

Within an agricultural context, (and again considering 2D × time analyses), the analysis of Trought and Bramley [48] considers the quality of grape juice by site across time. Their strategy is to fit different curves across time for each site, and then to look at spatial outcomes of their model by mapping. In considering longitudinal agricultural experiments, [49–52] and [53] use mixed models within a REML framework to analyse their spatio-temporal data, and fit state-space models via standard software and REML. The fixed part of their models is generally straightforward and the data are measured on two spatial dimensions.

Moving to three spatial dimensions and time, Higdon’s method of convolution with Gaussian kernels [13] allows for non-stationary spatial smoothing. The analyses of [54, 55] only give snapshots over time. Lemos et al [56], having failed to find much influence in terms of spatial proximity, model time sequences for each site using time-series methods. The convolutional approach is very attractive but possibly three dimensional spatial observations of ocean waters are smoother than soil observations over three spatial dimensions.

In an agricultural context, most studies involving three spatial dimensions and time do not attempt to model the data across all three spatial dimensions or across time. Thus. the soil profile study of [57] does not use spatial information in the analysis. Other studies composite the soils from different depths across soil types or treatment [58] thereby returning to a 2-dimensional × time analysis. However, Piepho et [49] present a four dimensional REML mixed modelling approach which is used by [59].

A major difference between agricultural data and epidemiological data which is so often modelled using the CAR prior of [8], together with an additive common spatially structured error, an additive common structured temporal random term (and an unstructured error with a variance common over both space and time), is that the spatial units of epidemiological data tend to vary slowly over time scales of a few years. Additionally, administrative time shocks may often be constant across a map, and hence this simple modelling structure works well. In contrast, the agricultural data modelled here vary markedly from sampling date to sampling date, and it is clear that the simple separable variance decomposition used by so many epidemiological models does not describe the data well.

### Modelling the case study data

In moving to four dimensions, there are many more possibilities for the decomposition of the fixed and residual parts of the model. Here, given the very different scales at which the spatial measurements were made, we chose to use CAR models for each depth layer, with neighbourhood smoothing occurring across the (roughly) horizontal layer. This choice to treat the third spatial dimension differently is made by others with three dimensional spatial data and for the same reason. Thus, Ridgway et al [60] separate out the depth component in their loess data fit, when modelling ocean temperatures.

For these data, the use of the CAR layered model [6, 7] also gave greater flexibility in modelling error components in the depth dimension, a flexibility which we found was needed. Thus, in the CAR model, depth is excluded depth from the neighbourhood error structures. (If depth neighbours were to be included as neighbours with equal weights, the horizontal layer information would be downweighted. If weighting uses functions of distance, the horizontal correlations would become effectively irrelevant.) This decision to define neighbours as neighbours only within the same depth layer gave the useful property that the CAR model is then permitted to have differing variances across the depths. For our model (Method 1, Eq 1), both the homogeneous and spatial variance components differ by depth, and while no formal tests were conducted, this flexibility in the model appeared better.

The Method 1 model is a date interaction model with the daily model. Each daily model is independent of each other which allows us to sum the DICs and the pDs [61, 62] over the 56 daily models and thus allows the possibility of a crude comparison of DICs with the models of Method 2, Eqs 6 and 8, where all 90,720 observations are modelled at once. We had planned to use this to compare the Method 3 models fitted to the full dataset, with the fit from the daily models from Method 1. However, the Method 3 models failed to converge, and hence, this was not done.

Method 1 gives appropriate 95% credible intervals for the contrast estimates, but no insight into the way in which these contrasts vary over time. The modelling strategy adopted in Method 2 attempts to remedy that by fitting time-varying covariates and by using time-series methods. Two-stage models do not account for the treatment effect variation observed in the model 1 fits. However they do allow us to see what level of complexity may be required to account for the time-varying nature of the contrasts.

A missing data model is shown in the supplementary materials (Fig L in S1 File). However, there is little point in fitting a missing data model when the posterior variance is largely dictated by the choice of a prior for a precision, as it must be with only 3% of the data being observed.

### Model Comparisons

Where competing models are suggested, the preference for model comparison is to use some summary statistic of the analysis fit such as the AIC [63] (used for geostatistical model comparisons by [64]), the BIC [65] or the DIC [29] which remains useful despite various criticisms [30]. When WinBUGS is used for model fitting, an obvious choice for a model comparison criterion is the DIC, which has the added advantage of estimating the number of parameters used by the model. Table 7 shows DICs for random walk models of order 1 where the only modelling difference is in the priors used for the two precisions. The differing priors make differences to the fit (shown by *R* ^2^) and to the DIC.

In arguing the case for the DIC, a CAR model is explicitly discussed in [29]. Their model has a CAR normal spatial prior, but the unstructured error component is Poisson, and therefore dictated by the estimates for the Poisson rate, which are themselves modified by the spatial CAR prior. For the agricultural data here, the CAR (and RW) models (both of which have two variance components) gave DICs which were highly sensitive to the choice of priors. The DICs of the autoregressive models are unaffected by the prior choice for the error, but the random walk models of order one often have estimates for the number of parameters which are greater than the number of observations used. Additionally, estimates for the number of parameters change with choice of prior, as does the DIC. This is not a problem of the criterion choice. Calculation of the BIC, which is also based on the final fit, gives essentially the same preferred models. The convolution prior of [8] which works so well for spatial epidemiological data, works less well when all the model components are normal and there is a single observation to be partitioned into structured and unstructured error, and a maximum of two neighbours, as is the case for the RW(1) models here. The random walk of order one with error partitioned into a normal random walk component and a normal observational error component is not identifiable. This generally does not lead to difficulties when the priors chosen for both precisions are identical, and sufficiently, but not overly diffuse. Here the partitioning was induced by restrictive priors on the random walk error, which had substantial influence on the model and led to very different model fits (Tables 3 and 5). However, random walks of order two with both observational and random walk error are identifiable, yet we still had problems with them. We believe that the RW(2) models gave unstable results as a consequence of the failure to standardise the outcome variable. When the outcome variable was standardised, the RW(2) models gave stable results for all sets of equal priors used. (These models, however, were not able to be compared with the earlier models via DIC or BIC.) Note that the DIC and its associated estimated number of parameters, pD, were extremely useful in alerting us to the problem of non-identifiability in the RW(1) models.

Our final view was that the purpose of the modelling across time was to develop insight into the time-varying nature of the contrast estimates. No ‘best’ model was chosen. Rather, the ‘better’ models with comparable DICS from Table 4, together with the daily penalised spline models and the Method 1 model were used to inform the description of the contrast of long fallowing versus response cropping. There is evidence of periodicity at the shallower levels (See Table 4) and this is also shown by the penalised spline smooths of Eq 6 and illustrated in Fig 5, with the double periodics per year at the shallower levels. At the depths of 200 cm and 220 cm there are fewer obvious peaks and troughs.

## Conclusions

Our purpose was to account for spatial and temporal autocorrelations in the context of four-dimensional data. The model of Method 1 and [7] forms the basis for the final analyses within this paper. It gave the fitted estimates for moisture at every combination of depth, date and treatment, and allowed a complex error structure, with an unstructured error at every depth, date and site, and with variances differing by depth and date. The spatially structured error was fitted across each horizontal layer and ignored depth neighbours. The variance of these structured spatial errors also differed by depth and date. Comparisons with three dimensional CAR neighbourhood models (not shown here) showed that that this separation of the two-dimensional plot arrangement from the depth dimension gave better descriptions of the data. See [66].

The simple expedient of fitting the data as a series of daily models allowed the maximum possible complexity in terms of the experiment and was a useful approach to modelling the full dataset. By fitting an interaction model by date at all levels of the daily model, we were able to explore more fully and more effectively the variability of the data, in particular, the variability of the variance components across the four dimensions. At the greater depths, seasonal variation in both contrasts and variances is less visible. See Fig 3 for the contrast, and the bottom panel of Fig 1 and the two lower panels of Fig 2 for the variances.

These analyses show that response cropping delivers lower moisture levels for most times of the year, in contrast to long fallow cropping. At the shallower depths, not surprisingly, this contrast exhibits considerable cyclicity which attenuates with depth. It appears that the temporal component adds little additional uncertainty to the contrasts which has not been captured by Model 1.

The method of defining neighbours within a horizontal layer has potentially wide applicability in three and four dimensional agricultural datasets, where the plot and treatment are defined by the two-dimensional surface coordinates. It may also be applicable in measurements made over the ocean where variables may also be measured at depth, again a situation where the differences in latitude and longitude between measurements far outweigh the differences in the depth dimension.

## Supporting Information

S1 File Supporting Figs A-AE. Figs A–X show point and 95% credible intervals for various models discussed in the text. Figs Y-AE show the estimated spatial and the unstructured variance components at the different depths over the period of the experiment.
(PDF)
