# Supplementary material for: A Four Dimensional Spatio-Temporal Analysis of an Agricultural Dataset
Source: PLoS One. 2015 Oct 29;10(10):e0141120. doi: 10.1371/journal.pone.0141120 (PMC4626095; doi:10.1371/journal.pone.0141120)
Supplement: S1 File — Figs A–X show point and 95% credible intervals for various models discussed in the text. Figs Y-AE show the estimated spatial and the unstructured variance components at the different depths over the period of the experiment. (PDF) [file pone.0141120.s001.pdf]

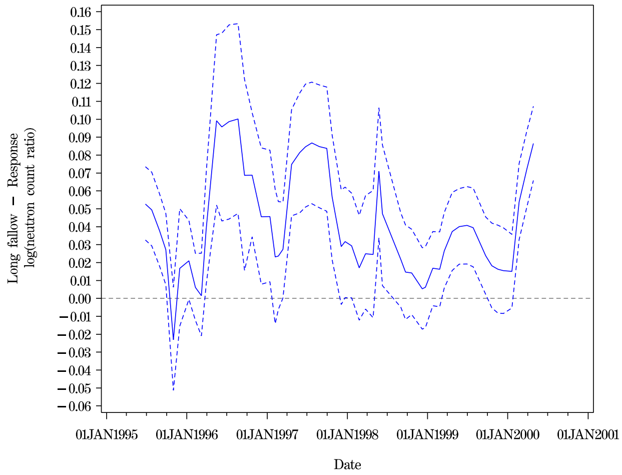

S1 Figure A. Long fallowing vs Response cropping at depth 100 for all trial days. Saturated model. Summary of MCMC iterates from the full model for the contrast. Estimates & 95% CIs.

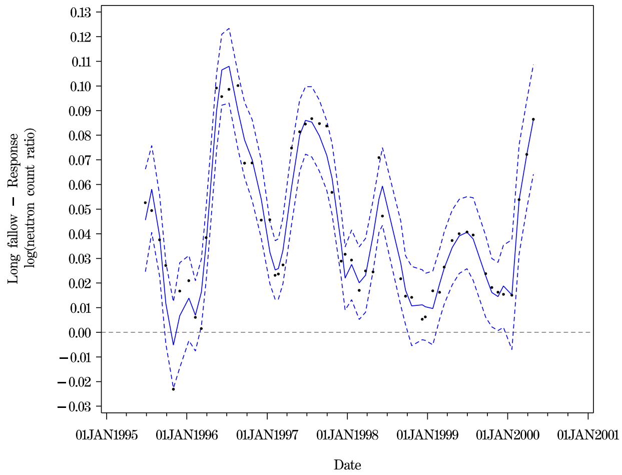

S1 Figure B. Long Fallowing vs Response cropping at depth 100 for all trial dates. Regression model (Equation 3.2) fitting 27 time-varying covariates. Point estimates & 95%CIs.

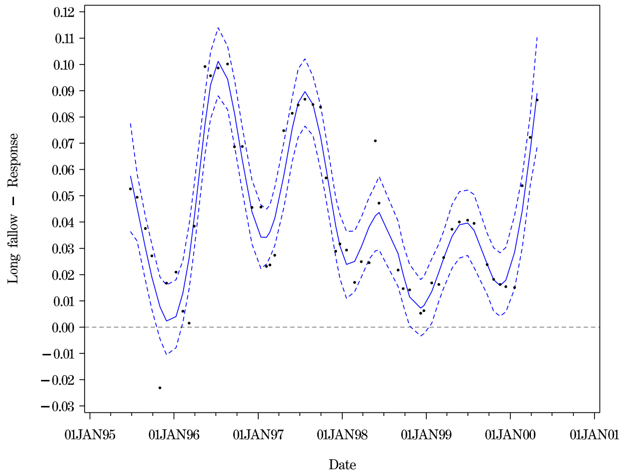

S1 Figure C. Long fallowing vs Response cropping at depth 100 cm for all trial days. Non-parametric penalised spline smooth across dates. Estimates & 95% CIs.

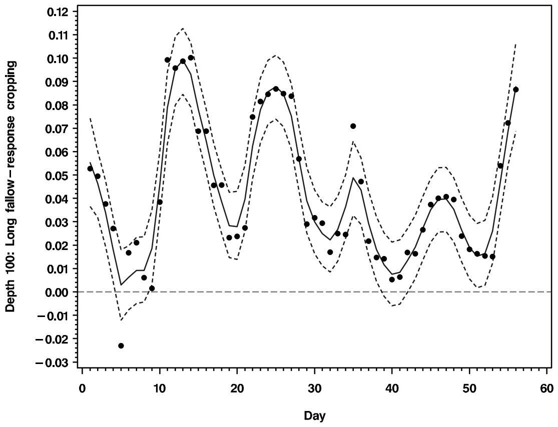

S1 Figure D. Long fallowing vs Response cropping at depth 100 cm for all trial days. Random Walk of order two. Estimates & 95% CIs.

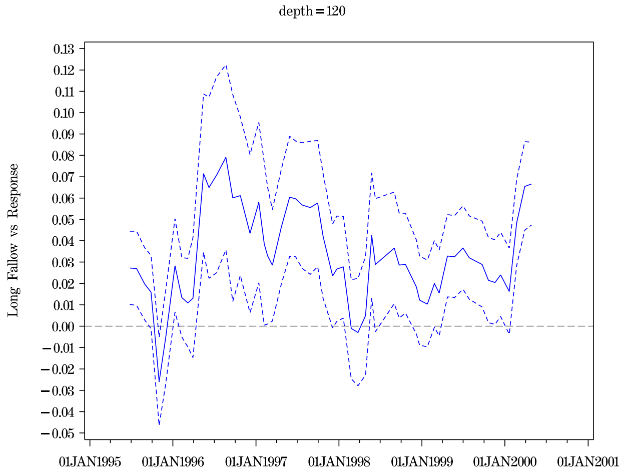

S1 Figure E. Long fallowing vs Response cropping at depth 120 for all trial days. Saturated model. Summary of MCMC iterates from the full model for the contrast. Estimates & 95% CIs.

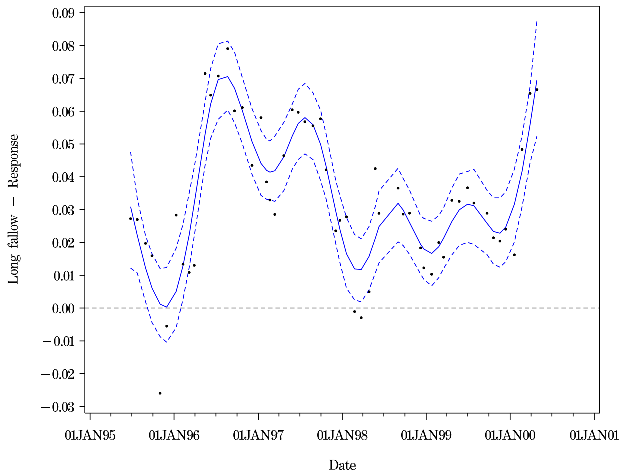

S1 Figure F. Long fallowing vs Response cropping at depth 120 cm for all trial days. Non-parametric penalised spline smooth across dates. Estimates & 95% CIs.

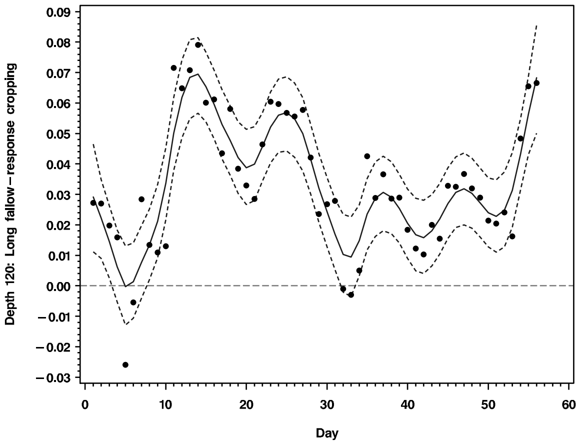

S1 Figure G. Long fallowing vs Response cropping at depth 120 cm for all trial days. Random Walk of order two. Estimates & 95% CIs.

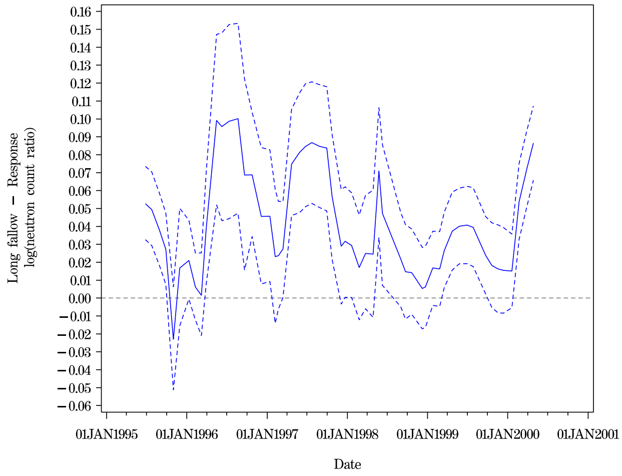

S1 Figure H. Long fallowing vs Response cropping at depth 140 for all trial days. Saturated model. Summary of MCMC iterates from the full model for the contrast. Estimates & 95% CIs.

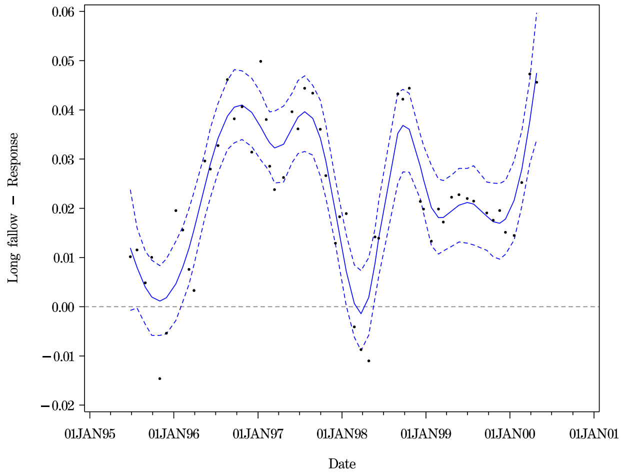

S1 Figure I. Long fallowing vs Response cropping at depth 140 cm for all trial days. Non-parametric penalised spline smooth across dates. Estimates & 95% CIs.

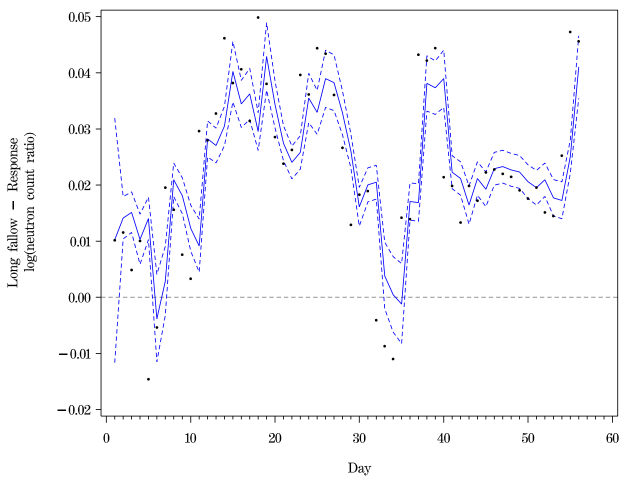

S1 Figure J. Long fallowing vs Response cropping at depth 140 for all trial dates (AR1 fit). Point estimates & 95%CIs.

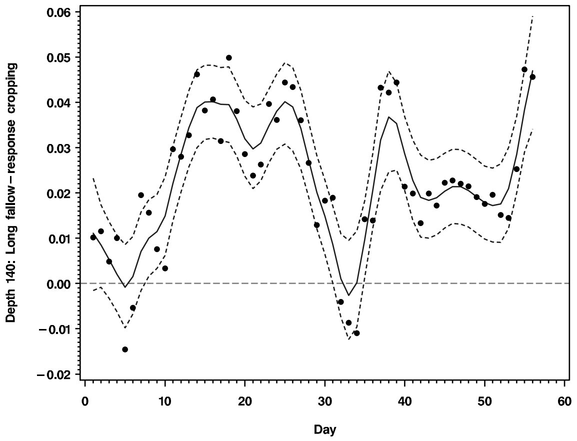

S1 Figure K. Long fallowing vs Response cropping at depth 140 for all trial dates (RW2 fit). Point estimates & 95%CIs.

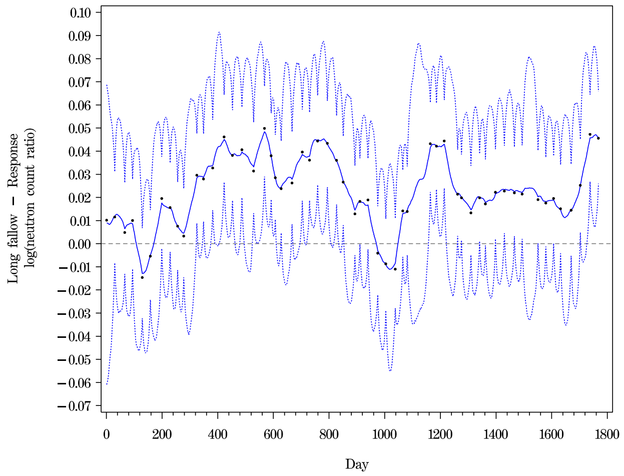

S1 Figure L. Long fallowing vs Response cropping at depth 140 for all trial dates. Random walk with 97% missing data. Random walk precision fixed at 2241 using Prior 3. See Table 1. Point estimates & 95%CIs.

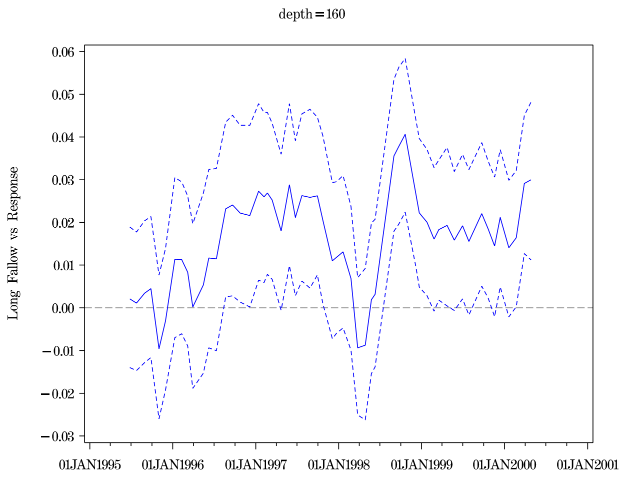

S1 Figure M. Long fallowing vs Response cropping at depth 160 for all trial days. Saturated model. Summary of MCMC iterates from the full model for the contrast. Estimates & 95% CIs.

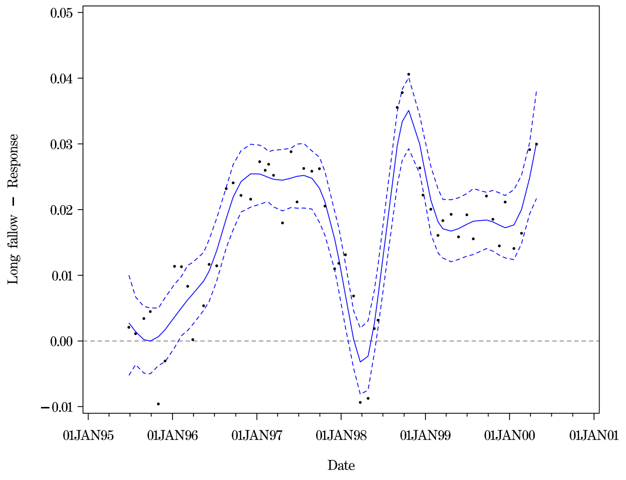

S1 Figure N. Long fallowing vs Response cropping at depth 160 cm for all trial days. Non-parametric penalised spline smooth across dates. Estimates & 95% CIs.

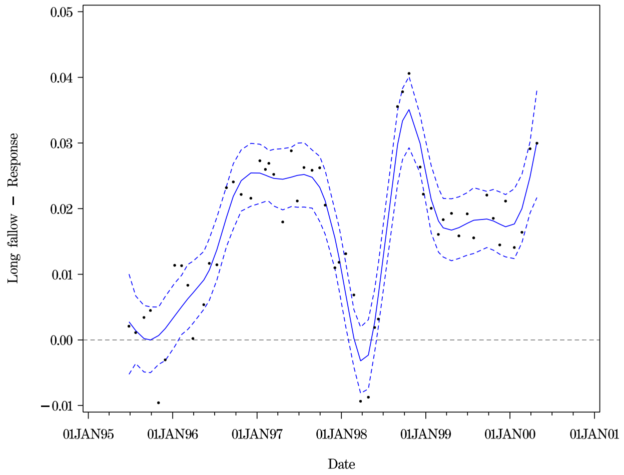

S1 Figure O. Long fallowing vs Response cropping at depth 160 cm for all trial days. Non-parametric penalised spline smooth across dates. Estimates & 95% CIs.

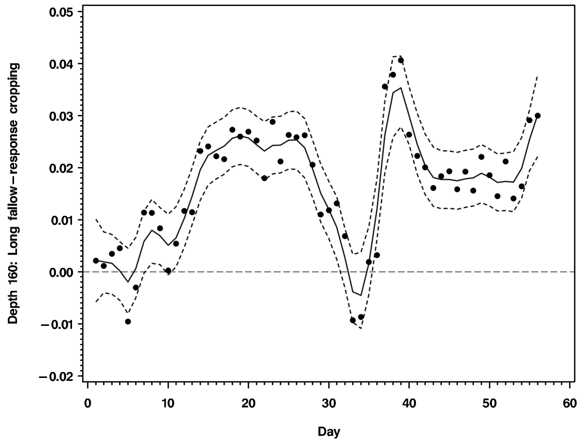

S1 Figure P. Long fallowing vs Response cropping at depth 160 for all trial days. Random Walk of order two. Estimates & 95% CIs.

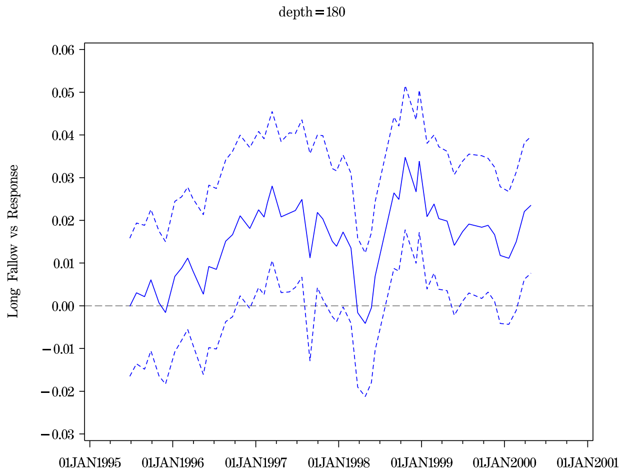

S1 Figure Q. Long fallowing vs Response cropping at depth 180 for all trial days. Saturated model. Summary of MCMC iterates from the full model. Estimates & 95% CIs.

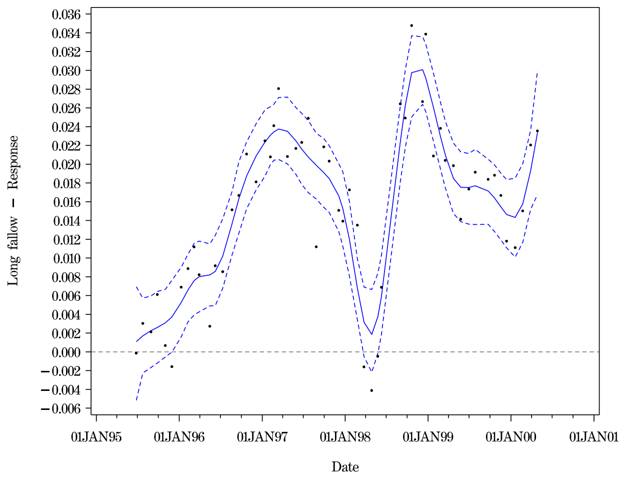

S1 Figure R. Long fallowing vs Response cropping at depth 180 cm for all trial days. Non-parametric penalised spline smooth across dates. Estimates & 95% CIs.

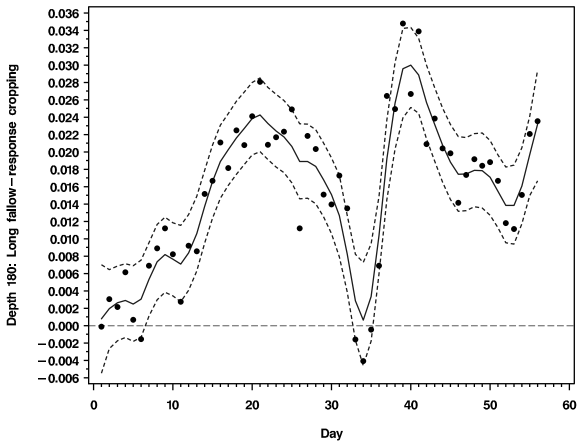

S1 Figure S. Long fallowing vs Response cropping at depth 180 for all trial days. Random Walk of order two. Estimates & 95% CIs.

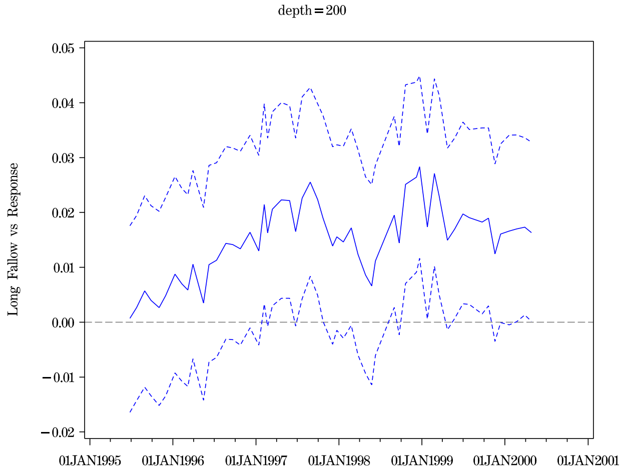

S1 Figure T. Long fallowing vs Response cropping at depth 200 for all trial days. Saturated model. Summary of MCMC iterates from the full model. Estimates & 95% CIs.

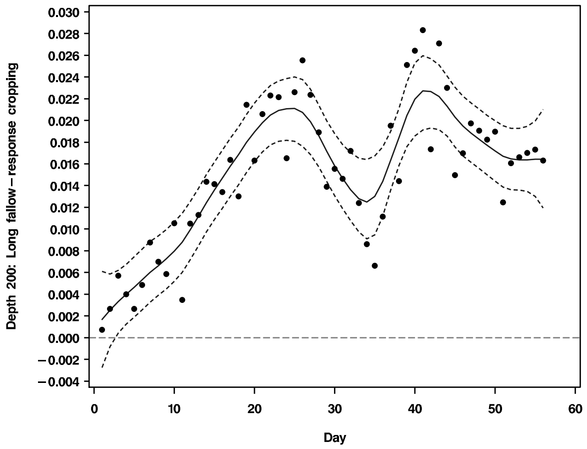

S1 Figure U. Long fallowing vs Response cropping at depth 200 for all trial days. Random Walk of order two. Estimates & 95% CIs.

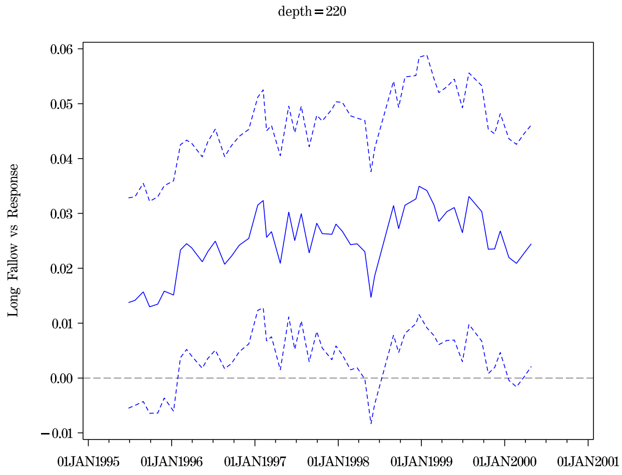

S1 Figure V. Long fallowing vs Response cropping at depth 220 for all trial days. Saturated model. Summary of MCMC iterates from the full model. Estimates & 95% CIs.

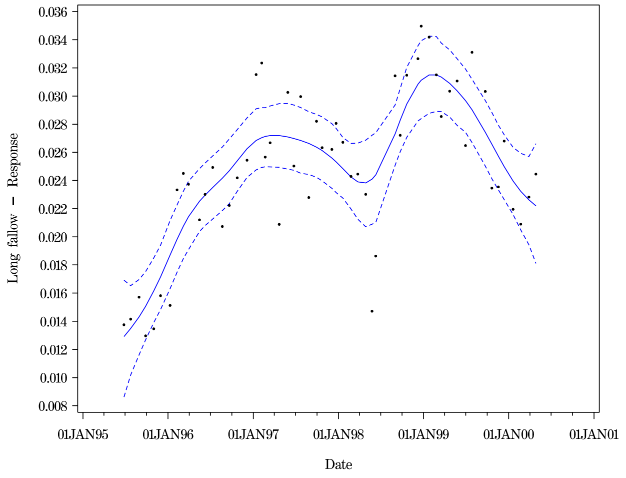

S1 Figure W. Long fallowing vs Response cropping at depth 220 cm for all trial days. Non-parametric penalised spline smooth across dates. Estimates & 95% CIs.

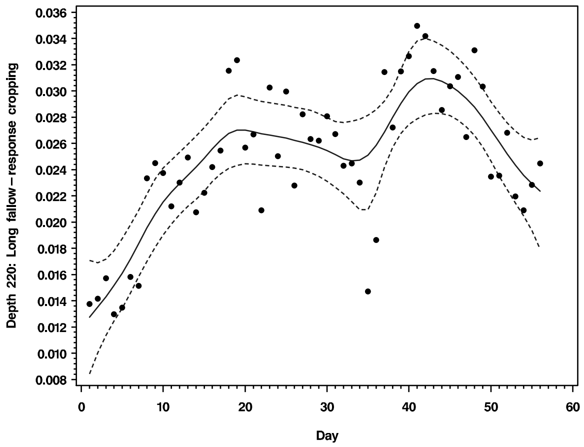

S1 Figure X. Long fallowing vs Response cropping at depth 220 cm for all trial days. Non-parametric penalised spline smooth across dates. Estimates & 95% CIs.

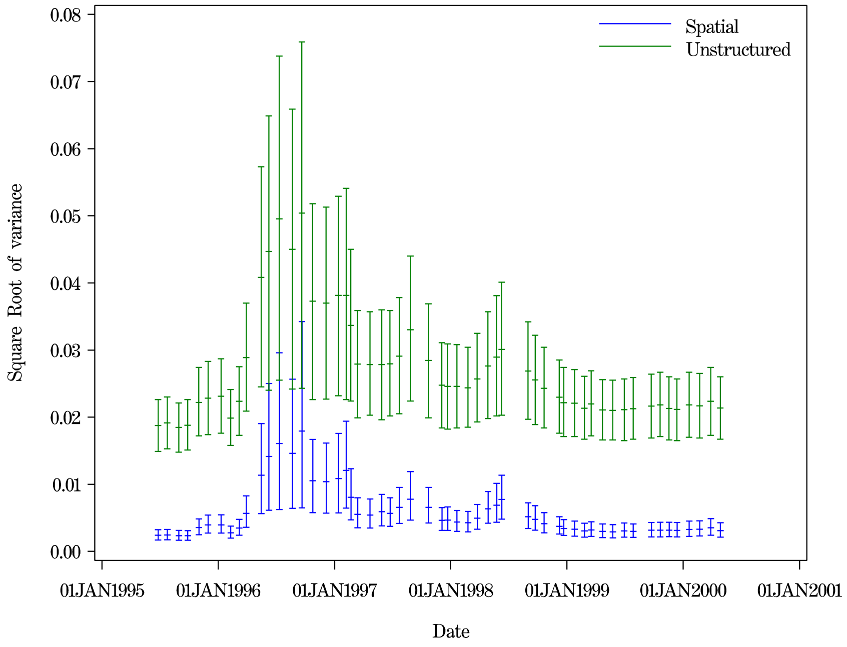

S1 Figure Y. Square root of variances & 95% credible intervals at depth 100 cm. Unstructured: green with broader bars, spatial: blue with narrower bars.

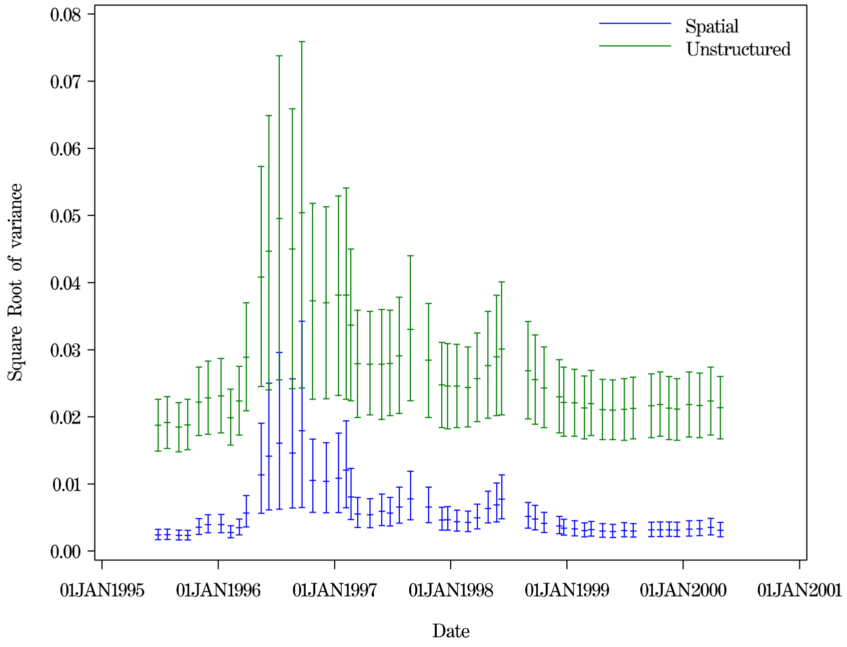

S1 Figure Z. Square root of variances & 95% credible intervals at depth 120 cm. Unstructured: green with broader bars, spatial: blue with narrower bars.

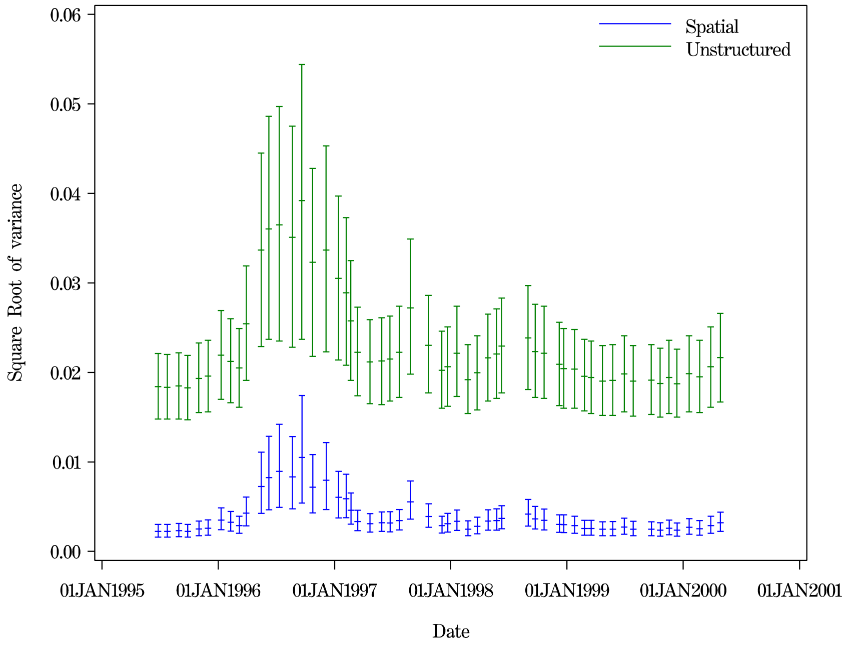

S1 Figure AA. Square root of variances & 95% credible intervals at depth 140 cm. Unstructured: green with broader bars, spatial: blue with narrower bars.

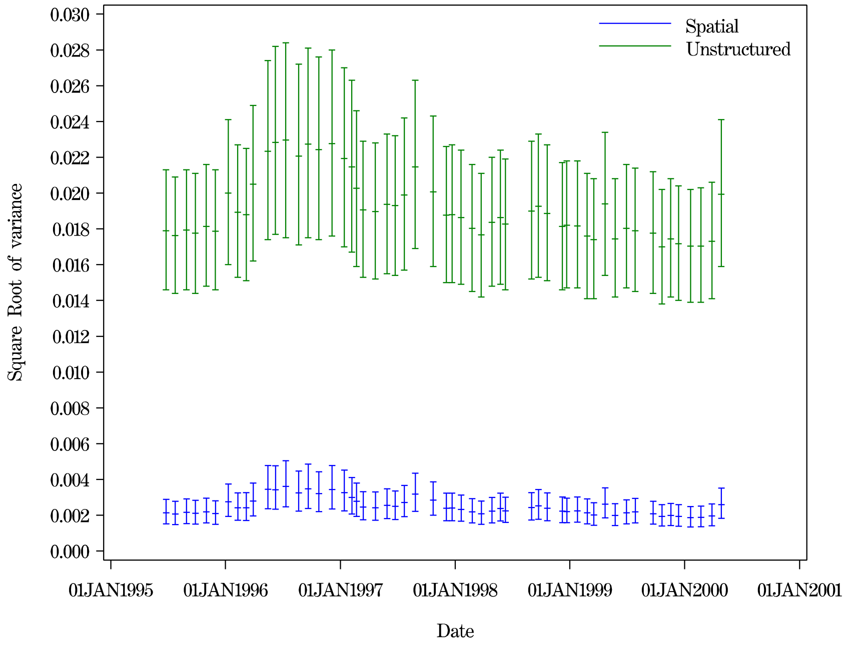

S1 Figure AB. Square root of variances & 95% credible intervals at depth 160 cm. Unstructured: green with broader bars, spatial: blue with narrower bars.

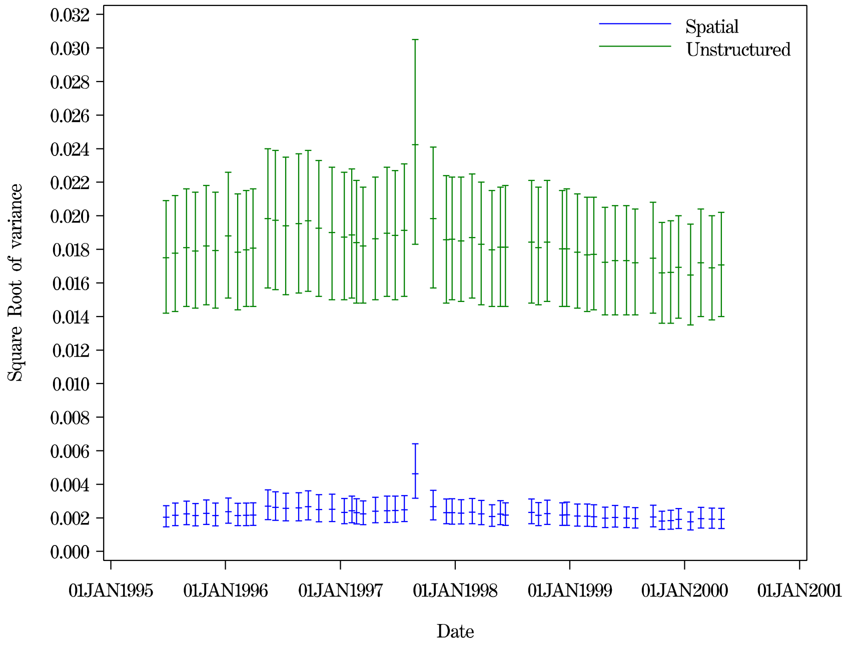

S1 Figure AC. Square root of variances & 95% credible intervals at depth 180 cm. Unstructured: green with broader bars, spatial: blue with narrower bars.

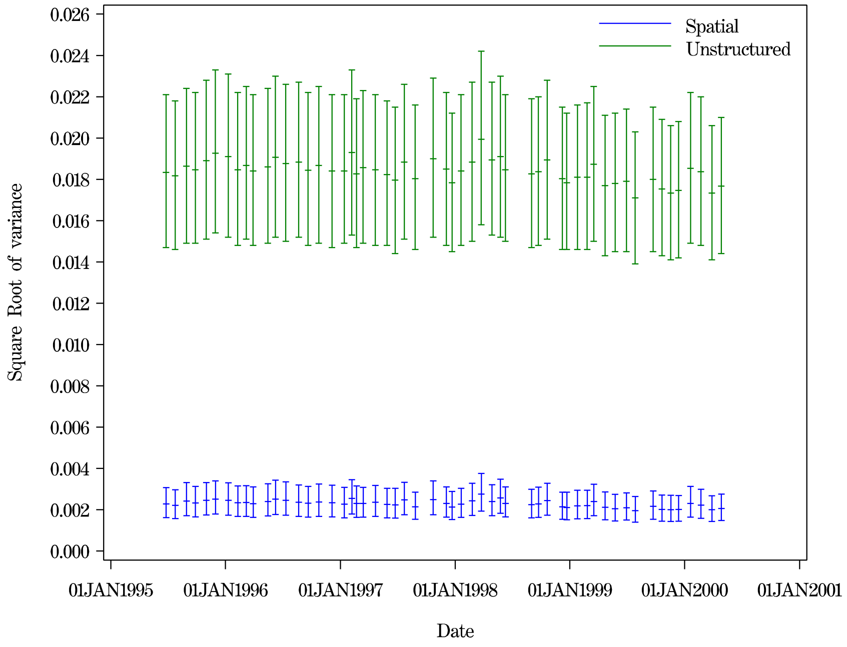

S1 Figure AD. Square root of variances & 95% credible intervals at depth 200 cm. Unstructured: green with broader bars, spatial: blue with narrower bars.

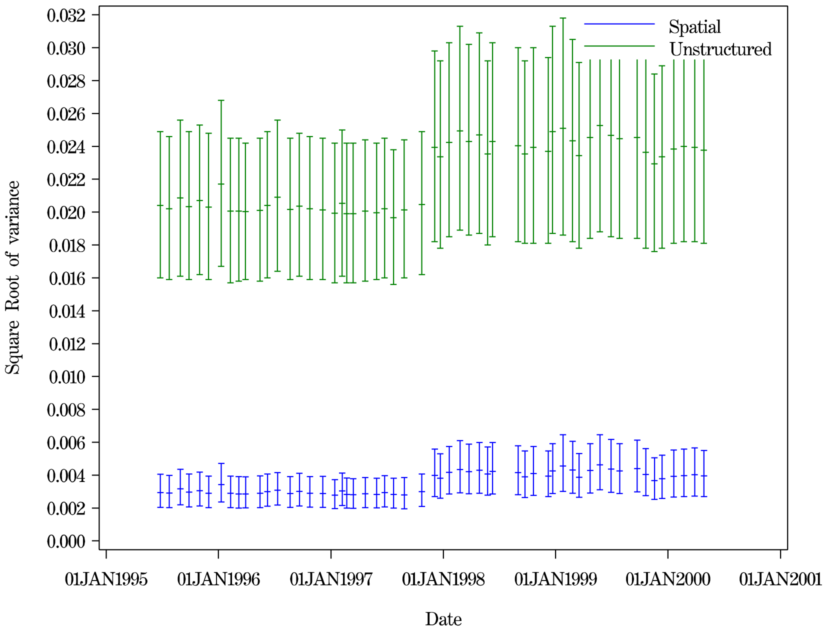

S1 Figure AE. Square root of variances & 95% credible intervals at depth 220 cm. Unstructured: green with broader bars, spatial: blue with narrower bars.
